# Capacity Building Efforts for Rabies Diagnosis in Resource-Limited Countries in Sub-Saharan Africa: A Case Report of the Central Veterinary Laboratory in Benin (Parakou)

**DOI:** 10.3389/fvets.2021.769114

**Published:** 2022-01-18

**Authors:** Fidelia Djegui, Morgane Gourlaouen, Andre Coetzer, Rachidatou Adjin, Rogatien Tohozin, Stefania Leopardi, Stephanie Mauti, Yao Akpo, Corneille Gnanvi, Louis H. Nel, Paola De Benedictis

**Affiliations:** ^1^Laboratoire de Diagnostic Vétérinaire et de Sérosurveillance de Parakou (LADISERO), Parakou, Benin; ^2^The Food and Agriculture Organization of the United Nations (FAO) and National Reference Centre for Rabies, The World Organisation for Animal Health (OIE) Collaborating Centre for Diseases at the Animal-Human Interface, Istituto Zooprofilattico Sperimentale delle Venezie, Viale dell'Università, Legnaro, Italy; ^3^Department of Biochemistry, Genetics and Microbiology, Faculty of Natural and Agricultural Sciences, University of Pretoria, Pretoria, South Africa; ^4^Global Alliance for Rabies Control SA Non-profit Company (NPC), Pretoria, South Africa; ^5^Department of Epidemiology and Public Health, Swiss Tropical and Public Health Institute, Basel, Switzerland; ^6^Faculty of Science, University of Basel, Basel, Switzerland; ^7^Direction des Services Vétérinaires, Cotonou, Benin

**Keywords:** rabies diagnostic, capacity building, surveillance, rabies elimination, phylogenetic analysis (phylogeny)

## Abstract

Rabies has been listed as a priority zoonotic disease in many African countries and the countdown to reach the goal of eliminating dog-mediated human rabies deaths by 2030 means that disease control measures need to be applied fast. In this context, an essential pillar of any national plan to control rabies is the implementation of reliable diagnostic techniques to ensure the success of field surveillance systems. Although many African countries have received international support for the control of rabies–some countries, like Benin, have not received a similar level of support. Indeed, until 2018, Benin was not able to diagnose rabies and rabies diagnosis in animals as well as humans relied solely on observed clinical symptoms. Although the Central Veterinary Laboratory (CVL) of Parakou had the equipment to implement two recommended tests, the lack of specific reagents and skills prevented the implementation of a rabies diagnostic service. Here we present the joint efforts of the national authorities in Benin, intergovernmental agencies, and non-governmental organizations to assess the strengths and weaknesses of the government's rabies control efforts. We have applied the Stepwise Approach toward Rabies Elimination (SARE) analysis, implemented rabies diagnostic capacities at the CVL of Parakou, characterized strains of rabies virus circulating in Benin, and finally integrated an inter-laboratory comparison program.

## Introduction

The most recent predictive disease burden models suggest that rabies, caused primarily by the *Rabies Lyssavirus* (RABV), causes an estimated 59,000 human deaths every year, which is likely an underestimation (1). As rabies has a higher burden on resource-limited countries in Africa—where disease surveillance and data reporting are often lacking or non-existent—it is often caught up in the cycle of neglect where the under-reporting of the disease results in rabies being given a lower priority by stakeholders and decision makers. This cycle of neglect can, however, be broken with the generation of empirical burden data that is generated through laboratory diagnosis and confirmation. In this context, improving the laboratory capacity of a country or a region ensures that animal rabies cases can be diagnosed efficiently, which in-turn ensures that (i) accurate data on the incidence of the disease is collected, (ii) control efforts can be monitored, and (iii) potentially exposed patients can be managed clinically. To this end, the network of centralized and decentralized diagnostic facilities that implement rabies diagnostic techniques that are in line with the recommendations of the World Organization for Animal Health (OIE) constitutes one of the main pillars of any rabies control plan and should thus be fostered where possible.

Through the contributions of the global community over the years, the rabies surveillance networks in various sub-Saharan countries have been supported, while other countries had yet to receive a similar level of support (2, 3). One of these countries in West Africa is Benin, which shares political borders with Burkina Faso, Niger, Nigeria, and Togo. Benin is a small country (~115,000 km^2^) with 11.5 million inhabitants that are spread out over 12 counties. Of those, ~265,000 people live in Porto Novo, which is the capital city located in the county of Ouémé. Although Benin is considered rabies-endemic and predictive burden models estimate that ~178 people die of preventable dog-mediated rabies every year, there is still very little data available regarding the actual epidemiological situation of rabies in the country (2). Indeed, data collected by the Public Health and Veterinary services between 2016 and 2019 indicated that 45 human rabies cases were diagnosed clinically. Furthermore, 1,978 dog bites and 77 canine rabies cases (diagnosed clinically) were officially reported between 2008 and 2018. While these cases were identified in nine of the twelve counties, the majority occurred in the Borgou County where the third largest city of the country, Parakou, is located.

Until 2018, the laboratory confirmation of rabies in either animals or humans was not available in Benin and diagnostic confirmation relied on the identification of clinical diagnosis, which is known to be misleading and inaccurate (4, 5). The Central Veterinary Laboratory (CVL) of Parakou, the Laboratoire de Diagnostic Vétérinaire et de Sérosurveillance (LADISERO), was built in 1985 thanks to the European Development Fund (EDF). However, due to a lack of further funding, diagnostic activities for diseases of animals (not rabies) only started in 1993 under the financial support of the Economic Community of West African States.

Here we present a collaborative effort between National Agencies in Benin and several International Organizations, leading to the advancement of Benin toward the overarching objective of the Zero-by-30 goal at a global level (6). First, a situational analysis of rabies in Benin was undertaken, after which the country's diagnostic capacity at the CVL was strengthened in various manners in response to the outcome of the landscape analysis. More specifically, in addition to establishing diagnostic confirmation for rabies at the CVL, we characterized recent canine strains of rabies virus circulating in Benin to gain an improved understanding of the epidemiology of rabies in the country. As pointed out in the study by Mbilo et al. (2), no scientific data on the rabies situation in Benin has been available to date. As such, to our knowledge, this is the first study on animal rabies in Benin presenting both a report of the actions undertaken to assist the government of Benin in eventually reaching the goal of zero dog-mediated human rabies by 2030 goal and a phylogenetic analysis of the canine rabies virus variants circulating in the country.

## Materials and Methods

### Stepwise Approach Toward Rabies Elimination Workshop

In January 2018 the Global Alliance for Rabies Control (GARC) undertook an in-country Stepwise Approach toward Rabies Elimination (SARE) workshop in collaboration with the Rabies Advisory Group of Benin as well as representatives from both the Ministries of Agriculture and Health. The SARE is a One Health oriented monitoring and evaluation tool that allows countries to undertake a comprehensive situational analysis of the rabies situation in their country. As a primary outcome, the SARE serves to highlight completed and pending activities that align with key components that make up a successful rabies elimination strategy, and provides an empirical score out of five, which is calculated based on the activities that have been completed according to the workshop participants (7–10). In addition to the SARE tool, the GARC Educational Platform (GEP) and the Rabies Epidemiological Bulletin (REB) was introduced during the workshop.

### Implementation of Multiple Diagnostic Tests at LADISERO

The implementation of diagnostic tests at LADISERO was guided by the availability of essential equipment and by the established previous experience of laboratory staff. Following the SARE assessment, the direct, rapid immunohistochemical test (DRIT) was implemented in Benin through a training workshop undertaken in 2018 at the Ministry of Health's National Service of Public Health Laboratories in Cotonou, Benin, relying on an established training methodology that had been used in various laboratories across Africa. Five local diagnosticians were trained in the implementation of the test, including two individuals from LADISERO in Parakou, one individual from the Veterinary Laboratory (LABOVET) in Bohicon, one private veterinarian from Cotonou, and one individual from the National Service of Public Health Laboratories in Cotonou. Over the course of the 3-day workshop, trainees were introduced to the DRIT assay and its use in a diagnostic setting by applying the assay to a cohort of brain samples that had been collected and stored specifically for the purpose of the workshop. In addition to the DRIT training course, the workshop also included a training on the safe and effective collection of brain samples by sampling through the occipital foramen to ensure an adapted sample collection methodology for diagnostic confirmation (11, 12) and the establishment of a rabies diagnosis database. Thereafter, any suspect rabies samples were delivered to the CVL for diagnostic confirmation using the DRIT assay as described elsewhere (13). During the routine implementation of the DRIT, each of the brain samples were blindly tested by treating the homogenized tissue impression smears with a biotinylated anti-ribonucleoprotein polyclonal antibody (PAb) preparation (ARC-OVI, Rabies Unit). Two experienced diagnosticians interpreted the results, and a consensus agreement was required before confirming the diagnostic outcome of any given sample.

Between March 2018 and June 2021, samples of central nervous system (CNS) tissues were collected from animals suspected to be infected with RABV. The samples were collected using the foramen magnum sampling procedure under strict biosafety conditions and procedures that aligned with the necropsy of potentially rabid carcasses. From the point of sample collection, the cold chain was maintained, and the samples were stored at −20°C at the CVL after being subjected to diagnostic confirmation (11, 14).

Due to a limited access of the laboratory to real-time RT-PCR technology, a conventional RT-PCR (15) was subsequently implemented at the CVL in Parakou through remote assistance in 2019. A French version of the detailed protocol was provided to the CVL via email (Supplementary Data). Briefly, each of the brain samples submitted at the CVL for rabies diagnosis were blindly tested by homogenization directly in lysis buffer and RNA was extracted using the RNeasy Mini kit (Qiagen). Analysis for the presence of lyssavirus viral RNA was performed (15) and amplicons were visualized using 1.5% agarose gel electrophoresis.

Finally, in February 2021, fifty Rapid Immunochromatographic Diagnostic Test (RIDT) kits [Rapid Rabies Ag Test Kit Bionote, Inc. (Hwaseong-si, Korea)] were also provided to the CVL in Parakou. Briefly, the cotton swabs provided in the kit were inserted directly into the brain samples to collect a pea-sized amount of brain tissue. The brain sample was placed into the buffer solution (provided in the kit) followed by thorough homogenization directly in the tube using the swab. Four drops of the buffer solution were then added to the sample inlet using a disposable dropper provided in the kit. The readout was made 10 min afterwards, as recommended by the manufacturers (16).

### Diagnostic Confirmation and Strain Characterization at an International Reference Center

A cohort of DRIT-tested samples (*n* = 10) were sent to the IZSVe for diagnostic confirmation. Of the ten samples, seven (7) were rabies-positive and three (3) rabies-negative by means of the DRIT assay as applied in Benin. Once delivered to the FAO RC for rabies, the samples were subjected to diagnostic confirmation by means of the DFA test and a conventional RT-PCR reaction as described elsewhere (15). Slides for the DFA test were prepared by smear technique, fixed, and then stained using a commercial anti-rabies nucleocapsid conjugate (Bio-Rad, 3572112) to which was also added some Evans blue counterstain (final concentration = 1%).

The products of RT-PCR were further characterized by Sanger sequencing before phylogenetic analyses were undertaken. Maximum likelihood (ML) nucleotide phylogenetic trees were inferred using PhyML (version 3.0) (17), employing the GTR+⌈4 substitution model, a heuristic SPR branch-swapping algorithm and 100 bootstrap replicates; obtained tree was edited online for graphical display using iTOL (18).

### Participation to an International Inter-laboratory Comparison

In the fall of 2020, the LADISERO, and sixteen other African laboratories, were invited to participate in an inter-laboratory comparison (ILC) test for the diagnosis of rabies. The sample panel to be tested included 12 freeze-dried blind coded samples and a starter kit composed of a positive and a negative control. Samples were prepared as previously described (19) following a well-established procedure. Prior to sending the samples, the vaccination status of the staff was controlled via the collection of a vaccination datasheet.

### Ethical Statement

The brain specimens were collected with the consent of pet owners or collectivities for stray animals. Animal health professionals are mandated to collect samples from non-endangered suspect rabid animals without requiring ethical permission since it is done under the framework of passive surveillance for rabies. The Animal Ethics Committee (AEC) of the University of Pretoria (South Africa) provided ethical approval for the implementation of the DRIT as described here (Approval numbers: EC027-16).

The samples prepared for the ILCs panel included (i) the use of laboratory animals and (ii) the use of samples collected and submitted to the laboratory and tested within the diagnostic activities carried out at the FAO Reference Center (RC) for rabies. (i) All experimental procedures involving laboratory animals were performed in strict accordance with the relevant national and local animal welfare bodies [Convention of the European Council no. 123 and National guidelines (Legislative Decree 26/2014)]. The Committee on the Ethics of Animal Experiments of the IZSVe approved the protocol (CE.IZSVe20/2014) which was then authorized by the Italian Ministry of Health (Decree 505/2015-PR). (ii) The brain tissue obtained from archived CNS samples of non-endangered mammals was collected in the framework of the national passive surveillance for rabies. All animals were found dead or legally euthanized by a competent veterinarian. According to the national legislation regulating animal experimentation, no ethical approval or permit was required for collecting and processing this type of specimen.

## Results

### In-country SARE Assessment in Benin

**Figure 1:**
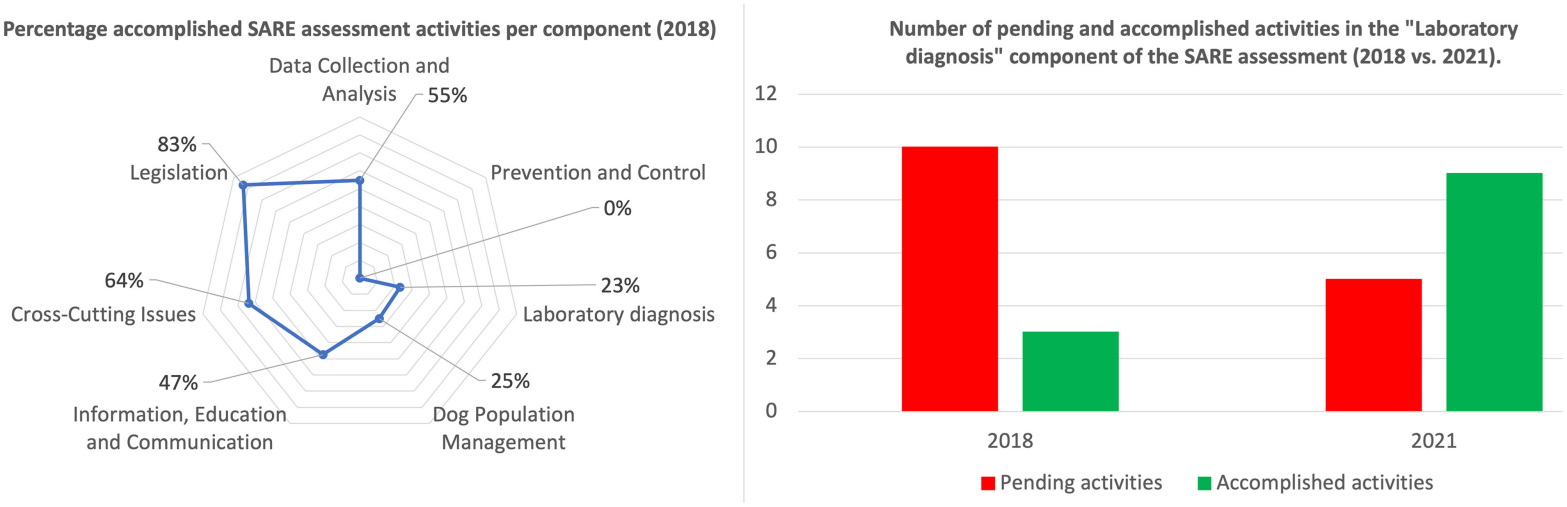
Summary of results for the SARE assessment of the national rabies control program, disaggregated by the seven components (left), and impact of the development of Benin's laboratory capacity (right).

After completing the SARE assessment, Benin's SARE score was determined to be 1.5 out of 5. This broadly signified that Benin had small-scale rabies control programs in place and that the government was working toward developing a national rabies control program. In addition to the score itself, the outputs generated by the SARE indicated that various SARE activities forming part of the different SARE components were still “pending” in Benin (Figure 1). Most significant in the scope of the work described here were the activities that aligned with laboratory capacity in Benin. More specifically, the participants indicated the following: (i) there was no laboratory testing of suspect rabid samples in the country (although diagnostic capacity was to be established after the workshop), (ii) there was no secondary diagnostic assay that could be used for diagnostic confirmation of suspect rabid samples, (iii) there was no participation in any proficiency testing initiatives with an internationally recognized laboratory and, (iv) there was no molecular characterization of RABV variants circulating in Benin (completed SARE output provided in Supplementary File 1). In support of the government's efforts to control rabies, these specific activities were addressed through various collaborative efforts described here (Figure 1).

### Rabies Diagnostic Flowchart Implemented at LADISERO Allows the Accurate Identification of Positive Samples

Between March 2018 and June 2021, 61 suspect rabid samples were subjected to diagnostic confirmation at LADISERO using the DRIT assay. The samples had all been collected from domestic animals, i.e., two (2) cats (2/61, 3.3%) and fifty-nine (59) dogs (59/61, 96.7%) (Table 1). Of these samples, 34.4% (21/61) were initially found to be rabies-positive (Table 1).

**Table 1:** Description of the samples analyzed throughout this study.

**ID number**	**Date of laboratory submission**	**Specie**	**Symptoms**	**Anamnesis (including putative origin/owner/history of vaccination)**	**Results dRIT test 1**	**Results DFA**	**Results RT-PCR at IZSVe**	**Results RT-PCR at CVL Parakou**	**Results dRIT test 2**	**Results LFD**
1	07/03/2018	Dog	Agitation, Salivation	Slaughtered for consumption at the discretion of the owner	Negative	N/A	N/A	Negative	N/A	N/A
2	29/03/2018	Dog	Agitation, Salivation	Slaughtered for consumption at the discretion of the owner	Negative	N/A	N/A	Negative	N/A	N/A
3	02/04/2018	Cat	Agitation	Biting animal/killed	Negative	Negative	Negative	Negative	N/A	Negative
4	04/04/2018	Dog	Hypersalivation	Slaughtered	Negative	N/A	N/A	Negative	N/A	Negative
5	11/04/2018	Dog	Agitation, salivation	Slaughtered for consumption at the discretion of the owner	Negative	N/A	N/A	Negative	N/A	N/A
6	21/04/2018	Dog	Agitation, salivation	Biting stray dog slaughtered immediately	Positive	N/A	N/A	Positive	N/A	Positive
7	19/05/2018	Dog	Agitation, salivation	Slaughtered for consumption at the discretion of the owner	Negative	N/A	N/A	Negative	N/A	N/A
8	09/05/2018	Dog	Abatement, agitation	Biting stray dog slaughtered immediately. No boost for vaccination	Positive	Positive	Positive	Positive	N/A	Positive
9	15/05/2018	Dog	Agitation, salivation	Slaughtered for consumption at the discretion of the owner	Negative	N/A	N/A	Negative	N/A	N/A
10	23/05/2018	Dog	Agitation, salivation	Slaughtered for consumption at the discretion of the owner	Negative	N/A	N/A	Negative	N/A	N/A
11	19/06/2018	Dog	Agitation, salivation	Slaughtered for consumption at the discretion of the owner	Negative	N/A	N/A	Negative	N/A	N/A
12	12/06/2018	Dog	Agitation, salivation	Slaughtered for consumption at the discretion of the owner	Negative	N/A	N/A	Negative	N/A	N/A
13	27/06/2018	Cat	Salivation	Biting cat immediatly slaughered. Non vaccinated	Positive	Positive	Positive	Positive	N/A	Positive
14	04/07/2018	Dog	Agitation, salivation	Slaughtered for consumption at the discretion of the owner	Negative	N/A	N/A	Negative	N/A	N/A
15	18/07/2018	Dog	Agitation, salivation	Slaughtered for consumption at the discretion of the owner	Negative	N/A	N/A	Negative	N/A	N/A
16	01/08/2018	Dog	Agitation, salivation	Slaughtered for consumption at the discretion of the owner	Negative	N/A	N/A	Negative	N/A	N/A
17	08/08/2018	Dog	Agitation, salivation	Slaughtered for consumption at the discretion of the owner	Negative	N/A	N/A	Negative	N/A	N/A
18	14/08/2018	Dog	Agitation	Biting animal slaughtered	Negative	N/A	N/A	Negative	N/A	N/A
19	23/08/2018	Dog	Agitation, salivation	Biting stray dog slaughtered immediately. Non vaccinated	Positive	Positive	Positive	Positive	N/A	Positive
20	28/08/2018	Dog	Salivation	Biting stray dog slaughtered immediately	Positive	Positive	Positive	Positive	N/A	Positive
21	11/09/2018	Dog	Salivation, agitation	Slaughered	Negative	N/A	N/A	Negative	N/A	N/A
22	20/09/2018	Dog	Prostation, apathy	Slaughered	Negative	N/A	N/A	Negative	N/A	N/A
23	15/10/2018	Dog	Agitation, hypersalivation	Biting animal slaughtered immediately	Negative	N/A	N/A	Negative	N/A	N/A
24	23/10/2018	Dog	Agitation, salivation	Slaughtered for consumption at the discretion of the owner	Negative	N/A	N/A	Negative	N/A	N/A
25	30/10/2018	Dog	Agitation, salivation	Slaughtered for consumption at the discretion of the owner	Negative	N/A	N/A	Negative	N/A	N/A
26	30/10/2018	Dog	Agitation, salivation	Slaughtered for consumption at the discretion of the owner	Negative	N/A	N/A	Negative	N/A	N/A
27	04/11/2018	Dog	Agitation, salivation	Slaughtered for consumption at the discretion of the owner	Negative	N/A	N/A	Negative	N/A	N/A
28	04/11/2018	Dog	Agitation, salivation	Slaughtered for consumption at the discretion of the owner	Negative	N/A	N/A	Negative	N/A	N/A
29	09/11/2018	Dog	Agitation, salivation	Slaughtered for consumption at the discretion of the owner	Negative	N/A	N/A	Negative	N/A	N/A
30	21/11/2018	Dog	Agitation, salivation	Slaughtered for consumption at the discretion of the owner	Negative	N/A	N/A	Negative	N/A	N/A
31	26/11/2018	Dog	Agitation, salivation	Slaughtered for consumption at the discretion of the owner	Negative	N/A	N/A	Negative	N/A	N/A
32	27/11/2018	Dog	Abatement, agitation	Biting stray dog slaughtered immediately. Non vaccinated	Positive	Positive	Positive	Positive	N/A	Positive
33	29/11/2108	Dog	Salivation	Slaughtered for consumption at the discretion of the owner	Positive	Positive	Positive	Positive	N/A	Positive
34	30/11/2018	Dog	Agitation, salivation	Biting stray dog slaughtered immediately. Non vaccinated	Positive	Positive	Positive	Positive	N/A	Positive
35	01/03/2019	Dog	Agitation, salivation	Slaughtered for consumption at the discretion of the owner	Negative	N/A	N/A	Positive	Positive	Positive
36	01/03/2019	Dog	Agitation	Biting animal slaughtered	Negative	Negative	Negative	Negative	Negative	Negative
37	18/07/2019	Dog	Agitation, salivation	Slaughtered for consumption at the discretion of the owner	Negative	N/A	N/A	Positive	Positive	Positive
38	18/07/2019	Dog	Salivation	Biting stray dog slaughtered immediately	Negative	N/A	N/A	Negative	N/A	N/A
39	19/07/2019	Dog	Agitation, salivation	Slaughtered for consumption at the discretion of the owner	Negative	N/A	N/A	Negative	N/A	N/A
40	27/07/2019	Dog	Agitation, salivation	Slaughtered for consumption at the discretion of the owner	Negative	N/A	N/A	Negative	N/A	N/A
41	27/07/2019	Dog	Hypersalivation	Biting animal slaughtered	Negative	N/A	N/A	Positive	Positive	Positive
42	27/07/2019	Dog	Hypersalivation	Biting animal slaughtered	Negative	N/A	N/A	Weak positive	Positive	Positive
43	27/07/2019	Dog	Agitation, salivation	Slaughtered for consumption at the discretion of the owner	Negative	N/A	N/A	Negative	N/A	N/A
44	27/07/2019	Dog	Agitation, salivation	Biting animal slaughtered	Negative	Negative	Negative	Negative	Negative	N/A
45	17/09/2019	Dog	Agitation, salivation	Slaughtered for consumption at the discretion of the owner	Negative	N/A	N/A	Negative	N/A	N/A
46	01/10/2019	Dog	Agitation, salivation	Slaughtered for consumption at the discretion of the owner	Negative	N/A	N/A	Negative	N/A	N/A
47	30/10/2019	Dog	Agitation, salivation	Slaughtered for consumption at the discretion of the owner	Negative	N/A	N/A	Negative	N/A	N/A
48	17/03/2020	Dog	Agitation, salivation	Slaughtered for consumption at the discretion of the owner	Positive	N/A	N/A	Positive	N/A	Positive
49	17/03/2020	Dog	Agitation	Biting animal slaughtered	Positive	N/A	N/A	Positive	N/A	Positive
50	17/03/2020	Dog	Agitation, salivation	Slaughtered for consumption at the discretion of the owner	Positive	N/A	N/A	Positive	N/A	Positive
51	17/03/2020	Dog	Salivation	Slaughtered for consumption at the discretion of the owner	Positive	N/A	N/A	Positive	N/A	Positive
52	28/05/2020	Dog	Agitation, salivation	Biting stray dog slaughtered immediately	Positive	N/A	N/A	Positive	N/A	Positive
53	28/06/2020	Dog	Anorexia, aggressiveness, and paralysis	Biting stray dog slaughtered immediately	Positive	N/A	N/A	Positive	N/A	Positive
54	28/06/2020	Dog	Aggressiveness, incoordination, salivation	Biting stray dog slaughtered immediately	Positive	N/A	N/A	Positive	N/A	Dubbious (EXCLUDED)
55	28/08/2020	dog	Aggressiveness, incoordination, salivation	Biting stray dog slaughtered immediately	Positive	N/A	N/A	Positive	N/A	Positive
56	29/08/2020	Dog	Salivation	Biting stray dog slaughtered immediately	Negative	N/A	N/A	Negative	N/A	Negative
57	19/09/2020	Dog	Anorexia, aggressiveness, and paralysis	Biting stray dog slaughtered immediately	Positive	N/A	N/A	Positive	N/A	Positive
58	19/09/2020	Dog	Aggressiveness, incoordination, salivation	Biting stray dog slaughtered immediately	Positive	N/A	N/A	Positive	N/A	Positive
59	21/09/2020	Dog	Anorexia, aggressiveness, and paralysis	Biting stray dog slaughtered immediately	Positive	N/A	N/A	Positive	N/A	Positive
60	24/12/2020	Dog	Aggressiveness, incoordination, salivation	biting dog	Positive	N/A	N/A	Positive	N/A	Positive
61	04/06/2021	Dog	Agitation, salivation	Biting animal slaughtered	Positive	N/A	N/A	Positive	N/A	Positive
Total negative samples					(40/61), 65.6%	/	/	(36/61), 59%	(36/61), 59%	
Total positive samples					(21/61), 34.4%	/	/	(25/61), 41%	(25/61), 41%	

*In red, the samples found negative by DRIT (test 1) but positive by RT-PCR. In blue, the samples sent to the IZSVe for confirmatory test analysis and submitted to phylogenetic analysis*.

**Figure 2:**
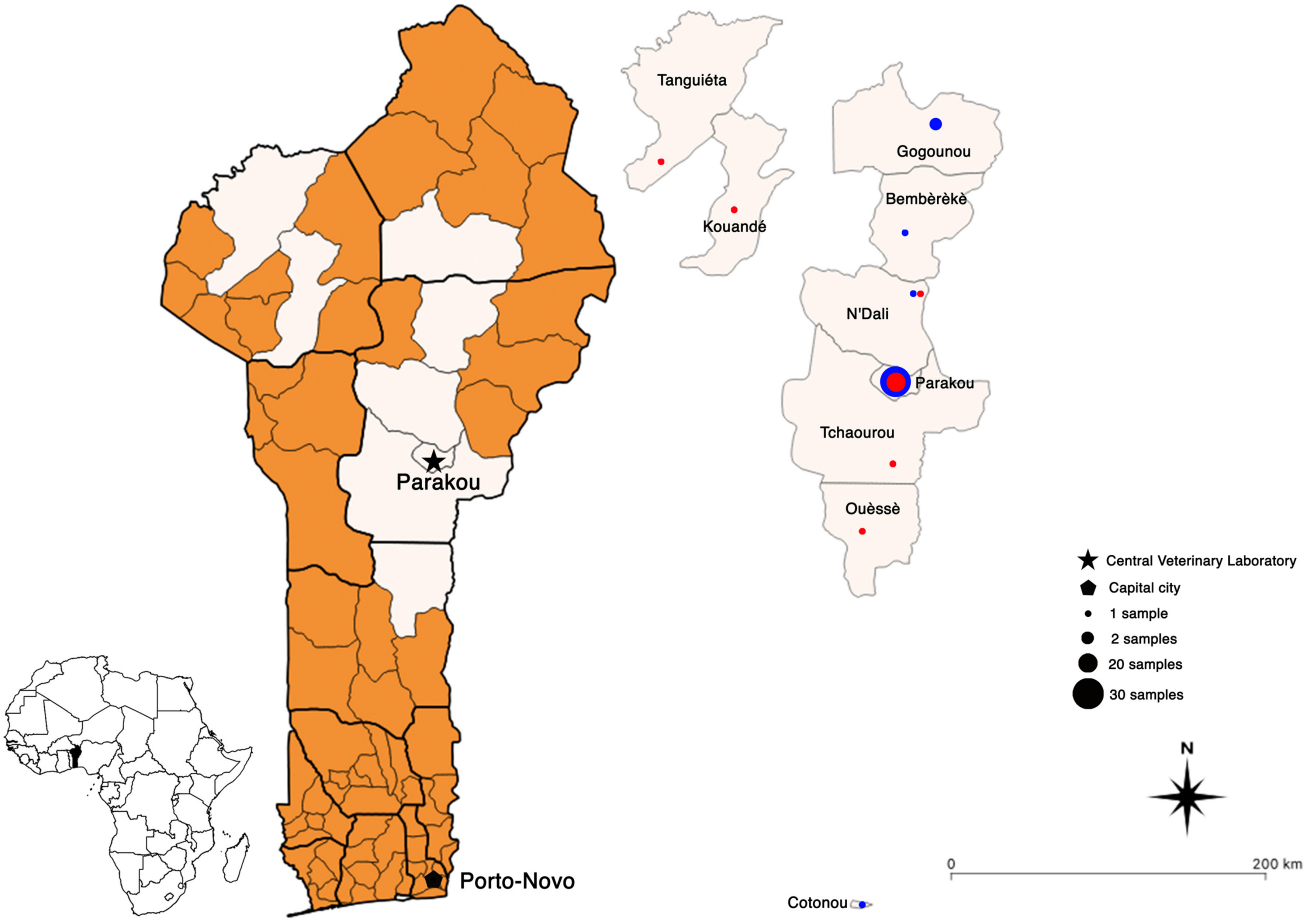
Geographical maps of Benin showing the location of all the rabies-positive (red dots) and -negative (blue dots) samples collected between 2018 and 2021. Map created using QGIS software (version 3.10).

The results obtained by nucleic acid amplification at the laboratory by means of the conventional RT-PCR showed that all 34 samples collected in 2018 matched those provided by the DRIT (Table 1). However, out of the 13 samples collected in 2019, 4 discrepant results were observed between the two techniques (DRIT and RT-PCR). As seen in Table 1, four samples (samples 35, 37, 41, and 42) were found rabies-negative by the DRIT, but rabies-positive when tested using RT-PCR. These four samples were blindly retested by means of the DRIT and results from the second test were rabies-positive. Therefore, twenty-five samples (25/61, 41%) tested positive for rabies after diagnostic confirmation was established by means of two diagnostic assays. A map presenting the geographical repartition of the collected samples is available in Figure 2.

To ensure that the diagnostic outcomes produced by the LADISERO were correct, ten samples (3 negatives and 7 positives) were shipped to the IZSVe for additional diagnostic confirmation in March 2019. Here, the samples were blindly re-tested by means of the DFA test as well as the same conventional RT-PCR protocol implemented at the CVL. All the results generated at the RC agreed with those from LADISERO, providing further evidence in support of the CVL correctly implementing the protocols (Table 1).

### Molecular Epidemiology of Rabies in Benin

**Figure 3:**
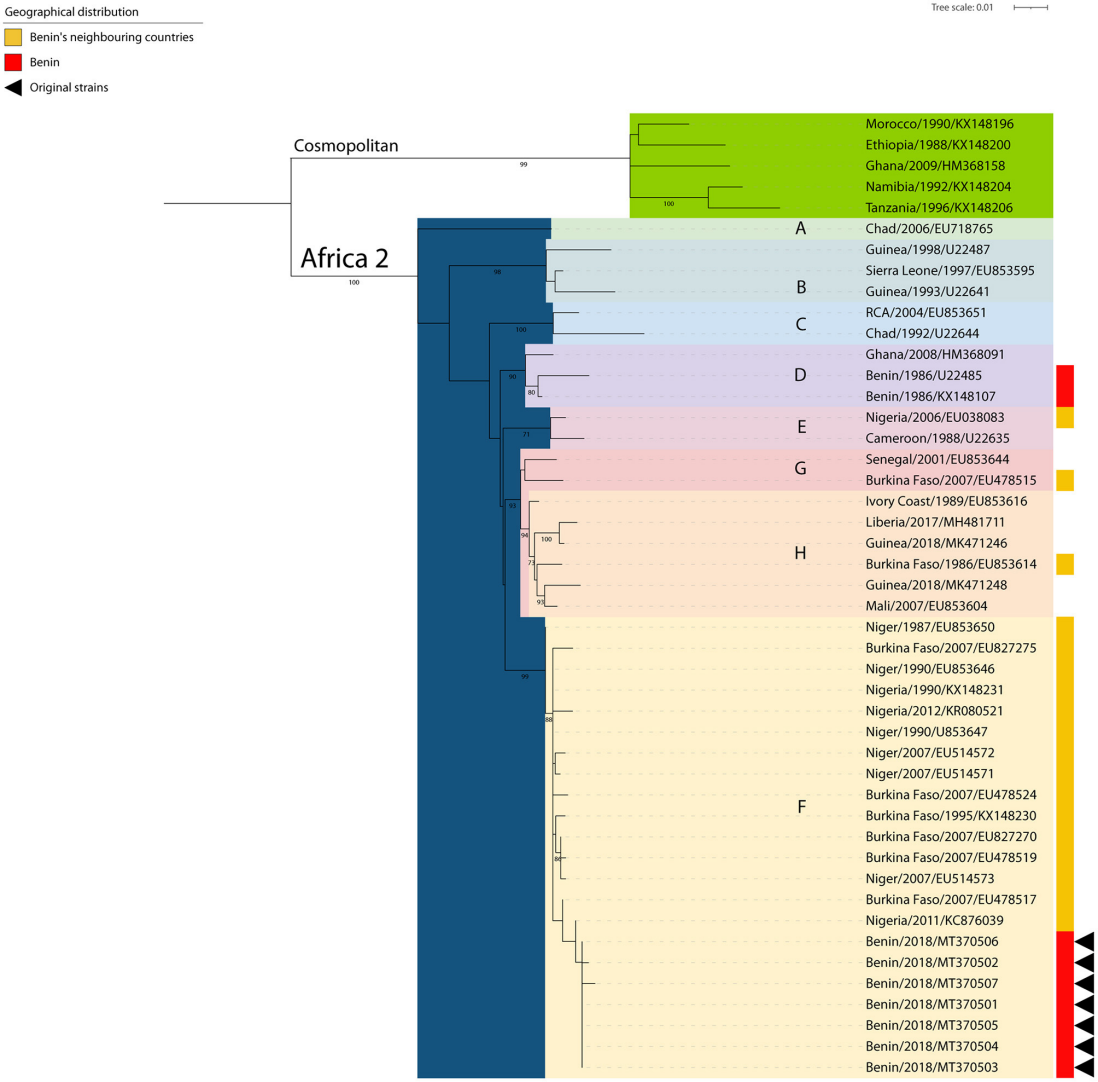
Phylogenetic tree constructed by Maximum Likelihood of the N gene. The rabies viruses from Benin are indicated with a red bar (right side) and those arisen from this study further identified with black triangles (right side). The rabies viruses collected from neighboring countries are indicated with a yellow bar (right side). SH-like supports higher than 70% are indicated next to the nodes.

To further enhance the resolution of the surveillance data, a phylogenetic analysis was performed on the seven (7) positive samples collected from dogs in Benin, i.e., Parakou (4/7), Tchaourou (1/7), N'Dali (1/7), Ouessé (1/7), and sent at the IZSVe for confirmatory analysis. The analysis of the seven (7) partial N gene segments showed that the RABV sequences circulating in 2019 in Benin belonged to the Africa 2 lineage, which is known to be circulating within terrestrial animal population in Central and Western African countries (20). More specifically, the new sequences clustered together with other viruses from the “F group,” circulating in neighboring countries such as Nigeria, Niger, and Burkina Faso (Figure 3) (20). The sequences were deposited in GenBank on the 23rd of April 2020 (Accession Numbers MT370501/MT370507).

### Implementation of Post-mortem Rabies Rapid Immunochromatographic Diagnostic Assays

All the positive samples (*n* = 25), and a small panel of negative samples (*n* = 4), received at the CVL were tested using the LFD after being subjected to diagnostic confirmation with the DRIT assay. The results showed 100% agreement in the detection of negative samples but very importantly, 100% agreement in the detection of the positive samples compared to the results obtained by the DRIT and the conventional RT-PCR (Table 1). One sample, sample ID 54, had to be excluded from the analysis as the device control line did not appear on two consecutive tests, despite the device's test line indicating a positive result in both instances.

### First Participation in an Inter-laboratory Comparison Test Confirmed the Appropriate Testing Procedure

Prior to receiving the panel of samples in November 2020, the vaccination status of the staff that were in contact with rabies suspected samples was checked and the three technicians in charge for rabies diagnostic activities at the CVL (Parakou) were found to be fully vaccinated against rabies. The CVL in Parakou tested the ILC panel by means of the DRIT and the conventional RT-PCR. In agreement with the acceptance criteria, the analysis of results demonstrated that the laboratory successfully passed this ILC and was able to correctly identify all the samples of the panel, thus demonstrating a high level of technical skills for the diagnosis of rabies. For confidentiality purposes, a detailed presentation of the results cannot be provided in this study.

## Discussion

The newly developed Global Strategic Plan for rabies elimination (GSP) (6) relies on a country-centric approach whereby governments need to take ownership of their national rabies programs; with the global community then identifying possible areas to assist and support national governments. In the case of Benin specifically, the Beninese government undertook an in-country SARE assessment in 2018 and identified various activities that would need to form part of their own national rabies control program. In so doing, the government outlined various activities that members from the global community identified as specific activities where support could be provided in furtherance of the national government's own efforts.

Prior to the work reported here, Benin was not able to diagnose rabies in either animals or humans due to a lack of specific reagents and/or skills required to implement any assays. This hindered the government's ability to showcase the burden of the disease and thus break the prevailing cycle of neglect. By establishing rabies diagnosis by means of the OIE-recommended DRIT assay [which involved the collaboration between the government, Swiss Tropical and Public Health Institute (Swiss TPH) and GARC] and confirmatory testing by means of a conventional RT-PCR (which involved the collaboration between the government and the IZSVe) in Benin, the government can begin to understand the disease burden on their population. In fact, the CVL had received and tested 61 samples in about 3 years—an incredible increase for a small and low populated country that had no capacity prior to the work presented here and out of which more than 40% were tested positive for rabies. Sadly, the number of samples received since 2018 has decreased significantly–thirty-four samples in 2018 and one sample in 2021. The reason for this is mainly attributable to the weakness of the surveillance system infrastructures currently in place in Benin, infrastructures that must be urgently strengthened along with the vaccination campaigns to achieve the final goal of Zero by 30. Indeed, the entire surveillance system was based on the CVL in Parakou field agents actively collecting samples in the field. Those agents who were specifically trained in the collection of field samples for the diagnosis of rabies, have been only partially included in a wider system and trained other staff, in order to lay the foundation of a solid and sustainable rabies surveillance. Recruiting and training field agents in Africa is not always an easy task, given many perceived or real competing disease priorities. In any case, the CVL in Parakou is also working to address this issue in the shortest delay and is currently establishing some collaborations with another division within the Veterinary Services. Although great achievement has been reached in Benin so far, the country's rabies epidemiology must be fed by continuous surveillance from the field. In this context, the role of operational veterinary services in collaboration with diagnostic laboratories is pivotal to ensure an efficient rabies control and prevention (21).

Furthermore, the CVL now has the capability to detect both the viral antigen and the viral nucleic acids, which dramatically decreases the chance of misdiagnosing samples. The importance of this ability to undertake diagnostic confirmation was highlighted in this study where the presence of four false negative results (two collected from biting dogs and two collected from dogs slaughtered for meat consumption) using the DRIT would have gone undetected had it not been for the use of the conventional RT-PCR. Importantly, these findings do not suggest that the DRIT is an inferior test or that it should be replaced by RT-PCR as both tests have pro and cons. The presence of false negative results by means of antigen detection can be due to various factors such as a weak viral load in the brain specimen collected for analysis, an inadequate quality of the brain specimen or a misinterpretation by the slide readers (22). Similarly, while the RT-PCR is more sensitive than antigen detection methods (both the DFA and DRIT), the risk of cross-contamination of the molecular approach is significantly higher in resource-limited laboratories (23). These results, as mentioned elsewhere (24), suggest that diagnostic confirmation by means of a second assay that is applied in parallel is of the utmost importance when confirming the presence or absence of rabies. Given the public health threat that animal rabies constitutes, the parallel analysis of high-risk suspect samples, principally those collected from biting animals or animals slaughtered for meat consumption, for the presence of the viral antigen and the viral nucleic acids should never be seen as a wasted effort and/or investment. Indeed, an ideal scenario for a laboratory possessing antigen and molecular detection platforms is to test any high-risk suspected samples either by DRIT or DFA test and to then confirm the results, both negative and positive, by means of RT-PCR.

Based on the collected data, 32 out of the 61 dogs (52.5%) were consumed after culling upon suspicion of rabies infection. Of those, 6 (18.8%) tested positive for rabies, including two dogs (sample 35 and 37) which were first tested as negative using the DRIT. Based on the information collected in the field, these animals were consumed before the diagnostic outcomes. Although the consumption of dog meat can be seen as taboo in some industrialized countries, it is a common practice in Sub-Saharan African countries with notably Nigeria often described as the largest consumer (25, 26). Given the prevalence of rabies in Africa, the risk associated for butchers, meat handlers, and consumers are high and should not be underestimated. As a matter of fact, studies which all took place in Nigeria showed that whilst butchers are not vaccinated against rabies, they frequently handle contaminated meat (25, 26). The level of positive attitude and practice related to a potential contact with rabies positive animals correlates with the level of education pointing out the need to raise awareness about the disease (27).

Overall, the samples collected near Parakou were sampled either at a meat market or at the Parakou veterinary clinic close to the CVL. Due to yearly awareness campaigns during World Rabies Day (WRD) which is celebrated on the 28th of September, the population of Parakou has adopted the appropriate behavior in case of animal rabies suspicion, i.e., to inform the local animal health services or to directly bring animals to the clinic. This further demonstrates the importance of correctly educating the population on the impact of the disease and the actions to take upon suspicion of animal rabies. Samples collected from other counties were processed by local animal health services and then sent to the CVL. Indeed, the lack of decentralized testing facilities implies that all samples must travel to the CVL for analysis using the DRIT and the RT-PCR. Although RIDT is not yet a recommended test for the detection of rabies antigens, it still constitutes a low technology diagnostic alternative which is also rapid, reader-friendly, and applicable to diagnostic screening under in-field condition. Within that framework, further evaluation of the performance of the RIDT could be highly beneficial in a country like Benin. The LADISERO received enough kits to test all the positive samples as well as a handful of negative samples. As a matter of fact, whilst the sensitivity of this type of kit is still under debate, their specificity has not been criticized. An updated protocol has been developed and so far, has proven to be highly performant in several studies (16, 28–31). In our hands, the results obtained using the RIDT were in total agreement with the ones obtained using the two gold standard techniques available at the LADISERO (Table 1). However, one sample had to be excluded, this sample resulted twice in being positive in the result test line but with the device control line not appearing. The exact reason behind this observation is unknown as this sample did not appear to be of a different texture or freshness of other tested samples. Importantly, GARC has recently developed the Rapid In-field Diagnosis and Epidemiology of Rabies (RAIDER) protocol which aims to facilitate the diagnosis of rabies in remote place with limited access to diagnostic facilities. The RAIDER protocol should further validate the use of the updated protocol for the RIDT (16) as a useful field screening approach thus facilitating fast responses when PEP is necessary of potentially exposed human cases. This protocol aims to promote the use of the RIDT as a screening method of samples directly on the field coupled with the shipment of samples to a laboratory applying a gold standard technique for final confirmation of rabies-status.

International travel of rabies suspected specimens is costly and requires the use of a dedicated courier under the Cat B UN2900 as defined by the IATA regulation. Thanks to the support of the Joint FAO/IAEA Center, the CVL was able to send 10 samples for diagnostic confirmation and further strain characterization to the IZSVe. Firstly, this collaboration established that sample analysis using the DRIT had been applied correctly by the CVL. Importantly, the appropriate application of the protocols and correct analysis of rabies suspected samples was further demonstrated by a successful participation in an inter-laboratory comparison (ILC) test in the fall of 2020. Secondly, the phylogenetic analysis of the seven (7) positive samples demonstrated that all of them belonged to the Africa 2 lineage/group F. This lineage of RABV is known to be circulating in neighboring countries (Nigeria, Niger, and Burkina Faso) (20), but this is the first time that a phylogenetic analysis focused on viruses collected from Benin is made publicly available. This finding, although expected, underlines the importance of monitoring the epidemiology of canine rabies circulation through a constant characterization of samples representative of the entire geographical area. Before our study, only one isolate collected from a cat in Benin in 1986 without any precise location (Accession Numbers U22485 and KX148107) was shown to cluster within the Africa 2 lineage/group D (20, 32). To this date, no information from Togo is available yet and overall sequencing from the entire region is occasional, thus jeopardizing any efforts in characterization. The conventional approach followed by partial sequencing selected herein provides a proof of principle that relevant epidemiological information can be obtained by the CVL in Parakou with a little additional funds. In this context, amplicons obtained at the CVL might be further sequenced making use of outsource facilities, thus enabling local authorities in assessing the origin of the circulating strains.

From the initial SARE assessment in January 2018 to the implementation of the DRIT as well as RT-PCR, the characterization of the strains circulating in Benin and the participation to an ILC, several milestones were achieved for the diagnosis of animal rabies in the country (Figure 1). Although partially supported by international partners (*per se* committed to capacity building in developing countries), all achievements were obtained thanks to the firm engagement of local staff. A summary of the costs related to the activities undertaken within the framework of the implementation of a rabies diagnostic service at the CVL of Parakou is described in this study and showed that about 16,000 euros (Supplementary Table 1) equivalent to about 19,000 US dollars was invested. This proves that even small investment can lead to great achievement, and this should be forwarded to decision makers and national authorities to improve the budget access.

## Data Availability Statement

The datasets presented in this study can be found in online repositories. The names of the repository/repositories and accession number(s) can be found at: https://www.ncbi.nlm.nih.gov/genbank/, MT370501/MT370507.

## Ethics Statement

The animal study was reviewed and approved by the Animal Ethics Committee (AEC) of the University of Pretoria (South Africa). Written informed consent was obtained from the owners for the participation of their animals in this study.

## Author Contributions

FD, MG, and PD conception and design of the study. MG and AC were involved in the manuscript drafting. FD, RA, RT, YA, and CG were all involved in the field study and collection of data. SL was involved in analysis of data. SM and LN were involved in the field study and implementation of methodology. All authors contributed to the article and approved the submitted version.

## Funding

This research was supported in part by a grant from the World Animal Protection (WAP) (Grant No. WAP/AF/CA/GR/2017-03), the Wolfermann Nägeli Foundation and the Swiss African Research Cooperation (SARECO).

## Conflict of Interest

The authors declare that the research was conducted in the absence of any commercial or financial relationships that could be construed as a potential conflict of interest.

## Publisher's Note

All claims expressed in this article are solely those of the authors and do not necessarily represent those of their affiliated organizations, or those of the publisher, the editors and the reviewers. Any product that may be evaluated in this article, or claim that may be made by its manufacturer, is not guaranteed or endorsed by the publisher.
