# Supplementary material for: Capacity Building Efforts for Rabies Diagnosis in Resource-Limited Countries in Sub-Saharan Africa: A Case Report of the Central Veterinary Laboratory in Benin (Parakou)
Source: Front Vet Sci. 2022 Jan 18;8:769114. doi: 10.3389/fvets.2021.769114 (PMC8805029; doi:10.3389/fvets.2021.769114)

STOP-R Technical

DATA COLLECTION AND ANALYSIS

Instructions: Enter "0" under Status if No or None, or "1" if Yes

STAGE		ACHIEVEMENTS / ACTIVITIES	OTHER IMPORTANT INFORMATION (please include in REMARKS)	STATUS	REMARKS	RABIES BLUEPRINT REFERENCES AND OTHER LINKS			
0	Surveillance	Does the national authority report at least one confirmed rabies case to the WHO, OIE or a regional rabies network?	Year of last reported rabies case; Examples of regional rabies networks include PARACON, ARACON and MERACON	1	Dec-17	WHO Collaborating Centres and OIE Reference Laboratories			
0		Have there been reports on rabies in the country?	e.g. media, newspaper, Twitter, Facebook, etc.	1	OIE Platform				
1		Are dog rabies cases reported from a local to the national level?		1	Revaping of the reporting system required	3.1.3-Infrastructure-surveillance	Challenges of animal health information systems and surveillance for animal diseases and zoonoses	Zoonotic diseases : a guide to establishing collaboration	6. Reporting of rabies data
1		Are human rabies cases reported from a local to the national level?		1		3.1.3-Infrastructure-surveillance	Challenges of animal health information systems and surveillance for animal diseases and zoonoses	Zoonotic diseases : a guide to establishing collaboration	6. Reporting of rabies data
1		Are you reporting all of the human or animal rabies cases that have been tested to a relevant international database such as WHO, OIE or a regional rabies network?		1		6.7 International databases			
1		Is there capacity to analyze dog rabies data at the national level?	For example, data analysis infrastructure in place for other diseases/programmes, adequately trained staff, sufficient technology, etc.	1		3.1.3-Infrastructure-surveillance	Challenges of animal health information systems and surveillance for animal diseases and zoonoses	2.3 Animal rabies surveillance	
1		Is there capacity to analyze human rabies data at the national level?	For example, data analysis infrastructure in place for other diseases/programmes, adequately trained staff, sufficient technology, etc.	1		3.1.3-Infrastructure-surveillance	Challenges of animal health information systems and surveillance for animal diseases and zoonoses	2.2 Human rabies surveillance	
1		Has an animal rabies surveillance* system been established at the national level?		1	Improve the operation	3.1.3-Infrastructure-surveillance	Challenges of animal health information systems and surveillance for animal diseases and zoonoses	Zoonotic diseases : a guide to establishing collaboration	2.3 Animal rabies surveillance
1		Has a human rabies surveillance* system been established at the national level?		1		3.1.3-Infrastructure-surveillance	Challenges of animal health information systems and surveillance for animal diseases and zoonoses	Zoonotic diseases : a guide to establishing collaboration	2.2 Human rabies surveillance
1		Are human bite case data collected and used for decision-making?		1		5.3.1-Rabies-surveillance			
1		Have dog population studies and KAP surveys been conducted to determine size, turn-over and accessibility of dogs for vaccination on a small scale?	Mention the years in which the studies were conducted	0		5.4.1-estimate the number of dogs			
2		Are human rabies surveillance systems, including feedback mechanisms, functioning and coordinated between administrative levels (national, province, district, municipal, etc.)?	Timeliness (e.g. monthly or real time) of reporting and feedback, feedback mechanisms established and stakeholders involved with clear reporting channels. (Follows on from establishment of data analysis capacity above)	1		5.1.1-epidemiology-of-rabies	WHO International Health Regulations		
2		Are animal rabies surveillance systems, including feedback mechanisms, functioning and coordinated between administrative levels (national, province, district, municipal, etc.)?	Timeliness (e.g. monthly or real time) of reporting and feedback, feedback mechanisms established and stakeholders involved with clear reporting channels. (Follows on from establishment of data analysis capacity above)	0		5.1.1-epidemiology-of-rabies	OIE Terrestrial Animal Health Code		
2		Have linked human and animal rabies surveillance systems, including agreed SOPs, been established?	Please describe the surveillance systems briefly, showing how data are shared. Note: Implementation of these linked measures is covered under 'Prevention and Control'	0					
2		Is information on the epidemiology of rabies regularly shared with all stakeholders?	Stakeholders involved - for example, policy makers, educators, NGOs and other relevant individuals and organisations	0		5.1.1-epidemiology-of-rabies	WHO International Health Regulations	OIE Terrestrial Animal Health Code	2. Roles-and-Responsibilities
2		Is the on-going surveillance system for rabies being maintained?	e.g. dog meat markets, opportunistic sampling (roadkill), human bite cases, etc.	1		WHO International Health Regulations	OIE Terrestrial Animal Health Code		
3		As your country nears human rabies elimination, have field investigations for all suspected human rabies cases been conducted?	Data shared between human and animal sectors in real time and joint actions taken	0		2.2 Human rabies surveillance			
3		As your country nears human rabies elimination, is epidemiological data being collected to provide evidence of dog-transmitted human rabies-free zones?		0					
3		Now that there are very few animal rabies cases in your country, are field investigations and laboratory confirmations conducted for all suspected rabies outbreaks in dogs?		0					
4		As there are no more laboratory confirmed human rabies cases, are existing surveillance activities for all suspected cases in humans maintained in the country?	e.g. clinical investigation of all patients presenting with encephalitic symptoms.	0					
4		As your country nears dog-rabies elimination, has the epidemiological data from the routine surveillance of all animals (not only dogs) been used to refine the national rabies strategy?	e.g. working animals, livestock and wildlife	0		WHO International Health Regulations			
5		Has freedom from dog-transmitted rabies in the entire country been verified by the absence of canine variant cases for at least a 2 year period?		0		5.4.17 Keeping an area rabies-free			

*DEFINITIONS:

Surveillance — Surveillance means the systematic ongoing collection, collation, and analysis of information related to human and animal health and the timely dissemination of information to those who need to know so that action can be taken. (Adapted from the OIE Terrestrial Animal Health Code 2010). It is the flow of data up to the national level and back down to the local level where activities are implemented.

STOP-R Technical

PREVENTION AND CONTROL

Instructions: Enter "0" under Status if No or None, or "1" if Yes

STAGE		ACHIEVEMENTS / ACTIVITIES	OTHER IMPORTANT INFORMATION (please include in REMARKS)	STATUS	REMARKS	Rabies Blueprint references and other links		
1	Human vaccines	Are vaccines for human rabies prophylaxis available in one or more parts of the country?		0		5.5.3-human-biologics	Human-vaccination-supply	
1		Has the supply and access to nationally licensed human rabies vaccines for PrEP for professionals at risk been ensured in local areas?		0				
2		Has an assessment* been done to determine the availability and access to PEP (and PreP)?		0		3.1-Infrastructure		
2		Are WHO pre-qualified human rabies vaccines available and accessible in most parts of the country?	Areas available and accessible	0		WHO expert consultation on rabies	WHO vaccines position papers	
2		Are any human biologics that are not WHO-pre-qualified being phased out? (e.g. nerve tissue vaccines, low quality vaccines)	If only WHO pre-qualified humans rabies vaccines are being used, mark the status as "1"	0		WHO prequalified vaccines list		
3		Are WHO pre-qualified vaccines and RIG available and accessible to high risk and exposed individuals throughout the country?		0		WHO prequalified vaccines list		
5		Now that your country is free from human and animal rabies, have modified protocols for PEP administration for rabies-free areas been implemented?	Modification of protocols includes improved risk-assessment before the administration of PEP, as the country is now free from endemic rabies.	0		WHO expert consultation on rabies		Guidelines on human prophylaxis
1	Dog vaccines	Are dog rabies vaccines available in at least one location in the country?		0		3.1-Infrastructure		
1		Has dog vaccination been initiated in some parts of the country?	Areas covered	0		5.4-What-are-we-going-to-do-dog-component		
2		Are only quality dog vaccines in accordance with OIE standards being used?		0		OIE terrestrial-manual		
3		Are mass dog vaccination campaigns reaching at least 70% of the total dog population conducted according to the plan described in the national rabies strategy?		0		5.4.13-Vaccination campaign-coverage		
3		Are post-vaccination surveys* in dogs being carried out to evaluate vaccination coverage?	Briefly describe post-vaccination survey	0		5.6-Evaluation		
4		Now that there are very few animal rabies cases in your country, are dog vaccination campaigns maintained in zones where dog rabies is still present or where otherwise justified (e.g. risk of introduction)?		0				
5		Even though you are rabies free based on successful mass vaccination campaigns, are strategic dog vaccination campaigns maintained where justifiable?	e.g. areas bordering endemic countries, ports of entry	0		5.4-What-are-we-going-to-do-dog-component		
1	Outbreak response and other rabies control activities	Have Standard Operating Procedures (SOPs) for coordinated action on reported outbreaks* at a local level been established?		0		Challenges of animal health information systems and surveillance for animal diseases and zoonoses		
1		Has Integrated Bite Case Management (IBCM)* been implemented at a local level?		0		Operational-activities		
2		Have IBCM SOPs, including sharing of information between sectors, been agreed upon at a national level?	Sectors involved. Please explain how the information is shared between sectors	0		WHO expert consultation on rabies	Challenges of animal health information systems and surveillance for animal diseases and zoonoses	Zoonotic diseases: A guide to collaboration
2		Is there active response to outbreaks in line with established SOPs?		0		5.4-What-are-we-going-to-do-dog-component		
2		Are SOPs available for the observation of rabies suspect dogs?	e.g. dogs involved in biting incidents	0		Guidelines-animal-shelters	WHO expert consultation on rabies	
2		Have facilities been established for the observation of rabies-suspected dogs?	Home quarantine is adequate so long as the owner ensures the dog is provided sufficient food, water and shelter during the observation period.	0		Guidelines-animal-shelters	ICAM animal shelter guidelines	
3		Is there capacity to conduct field investigations and planned outbreak responses for human rabies cases in the entire country?	Agencies of personnel involved in field investigations and outbreak response	0		WHO International Health Regulations	Challenges of animal health information systems and surveillance for animal diseases and zoonoses	
3		Have potential rabies-free zones been identified where animal rabies cases caused by the canine rabies variant have been absent for at least a 2 year period?	Names of rabies-free zones	0		5.4.17 Keeping an area rabies-free		
3		Has dialogue been initiated with neighbouring countries to prevent the re-introduction of rabies into designated rabies-free zones?		0		5.4.17-successful-maintenance	Challenges of animal health information systems and surveillance for animal diseases and zoonoses	
4		Is there capacity to conduct field investigations and planned outbreak responses for animal rabies cases in the entire country?		0				
4		Now that there are very few animal rabies cases in your country, has an emergency response/contingency plan been developed to address any reintroduced case of animal rabies involving a canine variant in rabies-free zones?		0		5.4.20 Reintroduction response		
5		Even though you are rabies free based on successful mass vaccination campaigns, has the capacity for outbreak and re-introduction response been maintained?		0		5.4.17-successful-maintenance		

*DEFINITIONS:

Assessment on access to PEP	Determination of the extent to which people can actually obtain post-exposure and pre-exposure treatment. This may differ from the availability of the vaccine, because other factors such as the cost and location of the
Post-vaccination surveys in dogs	Any survey conducted after a mass vaccination campaign has been implemented, and is used to determine the percentage of the dog population that has been vaccinated.
IBCM	Integrated Bite Case Management (IBCM) is a coordinated One Health approach to managing human dog bite cases, with the goal of improved communication and data sharing between the animal health and human health sectors following a bite case. It has a positive impact on animal rabies surveillance, identification of additional human exposures (bite cases), reduction in human rabies cases, and helps to target emergency canine vaccinations to areas where there are confirmed canine rabies cases.
Coordinated action on reported	When an outbreak is reported, the human and animal health services need to work in collaboration to ensure that it is investigated and dealt with effectively. SOPs should be created so that everyone understands their roles in an outbreak.

STOP-R Technical

LABORATORY DIAGNOSIS

Go to Dog Population Management

Instructions: Enter "0" under Status if No or None, or "1" if Yes

STAGE		ACHIEVEMENTS / ACTIVITIES	OTHER IMPORTANT INFORMATION (please include in REMARKS)	STATUS	REMARKS	Rabies Blueprint references and other links				
0	Specimen referral	Have contacts with an international rabies reference laboratory or international collaborating/reference center been established?	Names of international rabies reference laboratories or collaborating/reference centers	1	Istituto Zooprofilattico Sperimentale delle Venezie (IZSVe)	*3.1.7 Which laboratories are-available*	*OIE reference laboratories*	*WHO collaborating centres*	*FAO reference centre*	*WHO expert consultation on rabies*
0		If there is no laboratory diagnosis in your country, has at least one human or animal rabies suspect sample been submitted to an international rabies reference laboratory for confirmation?	If there has been any functional laboratory diagnosis in your country, mark this activity as "complete".<br>Year of most recent specimen referral;<br>Name of international rabies reference laboratory	1		*Simple-techniques-for animal brain-sampling*	*3.6 Human samples*			
1	Laboratory capacity and testing	Is rabies diagnosis being conducted in at least one national laboratory (veterinary or medical laboratory)?		1	Diagnostic capacity to be established following workshop	*3.1.8 Minimum laboratory requirements*	*OIE Manual of Diagnostic Tests and Vaccines for Terrestrial Animals*	*Laboratory biorisk management/*	*4. Laboratory rabies diagnosis*	
1		Have several rabies suspect samples of animals or humans been submitted to a national laboratory and analysed?	Name of national laboratory;<br>Laboratory test used	0	From February 2018	*Simple-techniques-for animal brain-sampling*	*Manual of Diagnostic Tests and Vaccines for Terrestrial Animals 2016*	*WHO guidance on regulations for the transport of infectious substances*		
2		Does your country undergo proficiency testing with an internationally recognised laboratory?	e.g OIE reference lab, WHO collaborating centre etc.	0		*6.7 International databases*				
2		Has capacity for regular sample collection and transportation been established and functioning?	Capacity includes the following: costing and payment system for sample transport, contact persons, transport companies/vehicles, packaging etc.	0		*OIE Manual of Diagnostic Tests and Vaccines for Terrestrial Animals*	*Laboratory biorisk management*	*5.3.1-Rabies-surveillance*		
2		Is there routine laboratory diagnosis of animal rabies cases in country?	e.g. if at least some suspect samples are diagnosed most (if not all) months of the year.<br>Names and locations of laboratories; Frequency of diagnosis	0		*3.1.8 Minimum laboratory requirements*	*Laboratory biorisk management*			
3		Is access to reliable laboratory diagnosis available throughout the country for animal samples (and if possible also for human and wildlife samples)?	Names and locations of laboratories	0		*3.1.8-laboratory diagnosis*	*4. Laboratory rabies diagnosis*			
3		Do you regularly assess your capacity to accurately diagnose suspect rabies samples (both human and animal)	e.g. diagnostic twinning, submission of samples to international laboratory, molecular confirmation (e.g. PCR)	0						
4		Now that there are very few animal rabies cases in your country, is there maintenance of existing surveillance activities, including ongoing laboratory investigation, for all suspected cases in dogs in the country?		0		*OIE Terrestrial Animal Health Code*				
4		Are terrestrial wildlife (carnivore) samples submitted for rabies laboratory diagnosis?		0						
4		Is there regular characterization and analysis of circulating rabies virus variants by a national or international laboratory?	Name of laboratory/institute where molecular tests are being performed	0		*OIE reference laboratories*	*WHO collaborating centres*	*WHO expert consultation on rabies*	*FAO reference centre*	
5		Even though you are rabies-free based on successful mass vaccination campaigns, are there on-going laboratory investigations of all suspected cases in domestic and terrestrial wild animal (carnivore) species in the country?		0		*OIE Terrestrial Animal Health Code*				

STOP-R Socio-cultural

DOG POPULATION RELATED ISSUES

Instructions: Enter "0" under Status if No or None, or "1" if Yes

STAGE	ACHIEVEMENTS / ACTIVITIES	OTHER IMPORTANT INFORMATION (please include in REMARKS)	STATUS	REMARKS	Rabies Blueprint references and other links	
1	Have discussions been held with stakeholders* to create a dog population management strategy at a local level?	Stakeholders involved, how this integrates with rabies control strategy. Studies and surveys from "Prevention and Control" need to be completed before this item.	0		*5.4.16 Dog population management tools*	*Guidelines-dog-population-management*
1	Have you involved officials in waste management in your stakeholder* meetings	Removal of garbage or preventing animals from reaching the garbage takes away available food sources for dogs. This will help to reduce the number of free-roaming dogs in the community.	0			
1	Has a baseline number of dogs been determined at a local level?		0			
2	Has a DPM* strategy and programme been drafted and shared with all relevant stakeholders at a local level?		0		*World Animal Protection-DPM guidelines*	*World Animal Protection-humane dog management*
2	Has the DPM strategy been finalized and implemented?	Please list what strategies are currently being used.<br>NOTE: Mass culling is NOT considered an effective DPM strategy. However, responsible dog management is considered appropriate. Euthanasia of suspect rabid animals according to the international standards is part of responsible dog management	0		*Monitoring and evaluation of dog population management programmes*	*OIE-terrestrial code chapter 7.7*
2	Has public sensitisation about DPM been built into rabies awareness campaigns at a local level?		1			
2	Have training or refresher courses on responsible dog management been initiated for professionals in animal health at a local level?		1		*GARC Animal Handling and Vaccination course*	
3	Has the DPM strategy been assessed and refined based on current dog ecology or KAP surveys done at a local level?		0	In some municipalities	*Knowledge-Attitude-Practice*	
3	Have rabies awareness campaigns, including responsible dog ownership, been expanded to more areas?	Intended audience and areas covered	1		*Communication-plan*	*5.4.16- dog-population-management*
3	Has veterinary and animal technician training been completed across most of country?		0			
5	Has the dog population management strategy been implemented nationwide?	Areas covered	0			
5	Have dog population management and responsible dog ownership campaigns been continued after the elimination of canine-mediated human rabies?		0		*Guidelines-dog-population-management*	

*DEFINITIONS:

Dog population management

Dog population management: Dog population management (DPM) is a multifaceted concept which aims to improve the health and wellbeing of free-roaming dogs and reduce problems they may present, within which permanently reducing the size or turnover rate of a dog population may be a goal (International Companion Animal Management Coalition, 2007). This goes far beyond dog population control which refers to a program with the aim of reducing a dog population to a particular level and/or maintaining it at that level and/or managing it in order to meet a predetermined objective (OIE, 2015a). Whilst the former is usually practiced using humane approaches and long-term objectives, the latter has often utilized inhumane methods with short-sighted goals.

DPM may be enacted for numerous animal welfare, public health or safety reasons associated with dogs, including human bite injuries, secondary infections and death, the spread of rabies and other zoonoses, noise and faecal contamination of the environment, traffic accidents, negative publicity for the government or an impact on tourism (International Companion Animal Management Coalition, 2007; Arluke and Atema, 2015; OIE, 2015a; World Animal Protection, 2015).

Therefore, DPM programs can have one or more goals depending on the specific situation, and will require a 'One Health' approach involving close collaboration between animal health, human health and environmental sectors. A DPM program with potential to improve animal, human and environmental health may increase motivation to tackle the issues and bring on board more stakeholders to support efforts.

(From: The Role of Dog Population Managementin Rabies Elimination – Current Tools andthe Need for Improvements. Taylor, LH et al)

Stakeholders

Examples include: Ministry of Health; Ministry of Finance; Mintry of Agriculture; Ministry of Education; Research/Training institutions (e.g. Universities); Local authorities (civil administration, police, environmental affairs); Local council; NGOs; Representation from international bodies (e.g. OIE, FAO, WHO, GARC, WAP)

STOP-R Socio-cultural

INFORMATION, EDUCATION, AND COMMUNICATION

Instructions: Enter "0" under Status if No or None, or "1" if Yes

STAGE		ACHIEVEMENTS / ACTIVITIES	OTHER IMPORTANT INFORMATION (please include in REMARKS)	STATUS	REMARKS	Rabies Blueprint references and other links	
1	Public awareness	Have the target audiences been identified at a local level (e.g. at-risk communities, dog owners, children)?		1		4.2.3 Understanding who needs to be involved	
1		Has an assessment been done to determine what message should be communicated to the target audience at a local level?	Determine who the target audience is and what message is appropriate to them. For example, through focus groups or surveys.	0		Examples of KAP surveys	
1		Has an IEC plan* been developed and implemented on a small scale?	Main messages and intended audiences (e.g. responsible dog ownership, rabies and bite prevention and management, and any other relevant messages for audiences such as dog owners, teachers, children, etc.)	1		Communication-plan	5.4.7-Awareness-campaign
1		Has broad public awareness messaging started at a national level?	To increase awareness about responsible dog ownership, rabies and bite prevention and management (through channels such as World Rabies Day, other health days, newspaper radio and television campaigns, and other channels)	1		World Rabies Day	
3		Has the IEC plan been integrated into the national rabies strategy, implemented at national level and updated where needed?	Main messages, intended audience and areas covered Consider including information to travellers	0		Communication-plan	
5		Have awareness programmes focusing on the maintenance of freedom from dog and dog-transmitted human rabies been implemented?	What are the main messages? Who is the intended audience? How often are messages broadcasted?	0			
1	Professional education	Have human and animal health professionals involved in rabies control been identified at a local level?		0			
1		Has an assessment been undertaken to determine the training needs of the professionals at a local level?	For example: Did officials assess whether doctors can diagnose rabies? Did officials assess whether veterinarians can vaccinate dogs safely?	0		5.3-Who-do-we-need-to-train	
1		Has a training plan been developed at a national level?		0		5.5-What-are-we-going-to-do-human-component	
1		Have training or refresher courses on rabies and public communication been initiated for professionals in human and animal health at a local level?	Participant organisations/agencies	1	DE. MSP. ORDRE. ABL-RAGE. SANOFI	GARC Education Platform	
2		Has training of human and animal health personnel been conducted in most parts of the country?	Participant organisations/agencies	1	ABL-RAGE. SANOFI	5.3-Who-do-we-need-to-train	GARC Education Platform
0	Advocacy	Is rabies a priority disease in your country?	This would be known after the disease prioritisation workshop has been undertaken in collaboration with the US Centers for Disease Control.	1		Public-health-and-economic-burden-of rabies	Health economic studies on rabies
1		Has an advocacy stakeholder analysis* been done at a national level and target audiences been identified?	For example, at-risk community leaders/authorities, politicians and other policy influencers and decision makers	1	ABL-RAGE		
1		Has an advocacy plan* been developed and implemented at a national level?	Include sharing of advocacy with neighbouring countries and regional level in national plan	1	Not implemented	Drafting an advocacy strategy	A toolkit for influencing policy
2		Have you promoted any small-scale rabies control program successes using the national advocacy plan?	Main messages, audiences, areas covered	0			
2		Has the national advocacy plan been used to obtain support from stakeholders to financially resource the national rabies control strategy?	Main messages, audiences	1			
3		Have dog-transmitted human rabies free zones been declared publicly?	Areas covered	0			
4		Have you declared publically that your entire country is free from dog-transmitted human rabies?		0			
5		Have you declared publically that your entire country is free from dog rabies?		0			

*DEFINITIONS:

IEC Plan — A structured plan for Information, Education and Communication (IEC) that has agreed objectives, audiences, messages, channels and timelines to increase awareness of rabies prevention and control. (See Blueprint links in SARE tool for guidance on creating a plan.)

Advocacy Plan — A structured plan to make rabies prevention and control a priority among leaders, decision-makers and policy influencers. (See Blueprint links for guidance on creating a plan.)

Advocacy stakeholder analysis — Identification of the people and organisations who play a role in rabies prevention and control, and determination of the most effective ways to help make rabies a priority for them. (See Blueprint links in SARE tool for guidance on creating a plan.)

CROSS-CUTTING ISSUES

Instructions: Enter "0" under Status if No or None, or "1" if Yes

STAGE		ACHIEVEMENTS / ACTIVITIES	OTHER IMPORTANT INFORMATION (please include in REMARKS)	STATUS	REMARKS	Rabies Blueprint references and other links		
0	Intersectoral collaboration	Are the results of rabies sample(s) shared with local and national authorities?	Agencies involved	0		*6. Reporting of rabies data*		
1		Have the main national stakeholders* in rabies prevention and control been identified?	Agencies of stakeholders involved	1	DE. MSP. ORDRE. ABL-RAGE. AMEVEP	*2. Roles-and-Responsibilities*	*Challenges of animal health information systems and surveillance for animal diseases and zoonoses*	
1		Are the key stakeholders* still actively involved in rabies control?		1	ABL-RAGE	*2. Roles-and-Responsibilities*		
1		Has an intersectoral rabies task force, committee or working group, including all relevant stakeholders*, been established at a local or national level and do they meet/communicate regularly?	Agencies of members of task force; methods of communication; regularity of meetings/communication	1		*2. Roles-and-Responsibilities*	*Challenges of animal health information systems and surveillance for animal diseases and zoonoses*	
2		Is the private sector included in the inter-sectoral task force's discussions and/or activities?		1		*2. Roles-and-Responsibilities*		
1	National programme and strategy	Based on a small-scale experience, has a short term rabies action plan been developed and endorsed by relevant authorities at local / national level?		0		*5.1-What-do-we-need-to-know-before*	*1.8-What-measures-are-available*	*2. Roles-and-Responsibilities*
1		Have mechanisms for mobilizing emergency funds for rabies control been identified?		1		*3.3-Costs-and-Funding*		
2		Has a national strategy for rabies prevention, control and eventual elimination been drafted and finalised in collaboration with all relevant stakeholders?		1		*Components-of-a-successful-rabies-programme*		
2		Have government resources been identified and allocated in support of the national rabies control strategy?		1	Insufficient resources	*3.3-Costs-and-Funding*		
3		Now that there are very few animal rabies cases in your country, has the national strategy been refined based on current data?	e.g. molecular epidemiological information, current research studies/surveys, PEP usage and bite case data.	0		*5.6-Evaluation*	*5.7.1-Sustainability*	
4		Have veterinary border inspection and quarantine measures been fully implemented in accordance with national regulations?		0		*OIE Terrestrial Animal Health Code*		

*DEFINITIONS:

Stakeholders: Examples include: Ministry of Health; Ministry of Finance; Minstry of Agriculture; Ministry of Education; Research/Training institutions (e.g. Universities); Local authorities (civil administration, police, environmental affairs); Local council; NGOs; Representation from international bodies (e.g. OIE, FAO, WHO, GARC)

LEGISLATION

Instructions: Enter "0" under Status if No or None, or "1" if Yes

STAGE		ACHIEVEMENTS / ACTIVITIES	OTHER IMPORTANT INFORMATION (please include in REMARKS)	STATUS	REMARKS	Rabies Blueprint references and other links			
0	Animal rabies national case definition	Is a case definition available that is consistent with the OIE standards for animal rabies?		1		*OIE Terrestrial Animal Health Code*			
1		Has the case definition for animal rabies been shared with all of the relevant professionals?	Agencies the case definition was disseminated to	1		*OIE Terrestrial Animal Health Code*			
0	Human rabies national case definition	Is a case definition available that is consistent with the WHO guidelines for human rabies?		1		*WHO expert consultation on rabies*			
1		Has the case definition for human rabies been shared with all of the relevant professionals?	Agencies the case definition was disseminated to	1		*WHO expert consultation on rabies*			
1	Legal Framework	Is there national legislation that is relevant to rabies prevention and control?	For example: an Animal act; compulsory vaccination, etc.<br>Title of the framework and the year it was passed	1		*3.2. Legislation*			
1		If there is legislation, has it been reviewed and endorsed?	Year the framework was reviewed	0		*3.2. Legislation*			
1		Has rabies been made a notifiable disease in animals?		1		*3.2. Legislation*	*3.2.3-Why-does-rabies-need-to-be-notifiable*	*OIE Terrestrial Animal Health Code*	*3.2.9-How-to-make-rabies-notifiable*
1		Has rabies been made a notifiable disease in humans?		1		*3.2. Legislation*	*3.2.3-Why-does-rabies-need-to-be-notifiable*	*WHO expert consultation on rabies*	*3.2.9-How-to-make-rabies-notifiable*
1		Does legislation include measures for rabies outbreak response?		1					
2		Does the legislation regarding nationally licenced vaccines reflect that of international guidelines?		1					
3		Has legislation been updated to include specifications on the compulsory vaccination and international movement of dogs?		0		*General-guide-on-veterinary legislation*	*3.2.11- laws-and-by-laws*		
3		Is the relevant legislation enforced at the national level?		1					

Stepwise Approach Towards Rabies Elimination - Benin

STAGE 1,5

ACTIVITY SUMMARY

COMPONENTS	PENDING ACTIVITIES	ACCOMPLISHED ACTIVITIES
**Data collection and analysis**<br>*Total number of activities = 22*	10	12
**Prevention and Control**<br>*Total number of activities = 26*	26	0
**Laboratory diagnosis**<br>*Total number of activities = 13*	10	3
**Dog population related issues**<br>*Total number of activities = 12*	9	3
**Information, Education, Communication**<br>*Total number of activities = 19*	10	9
**Cross-cutting issues**<br>*Total number of activities = 11*	4	7
**Legislation**<br>*Total number of activities = 12*	2	10

STAGE SUMMARY

STAGE*		PENDING ACTIVITIES	ACCOMPLISHED ACTIVITIES	STAGE COMPLETED?
0	*Total number of activities= 8*	1	7	COMPLETED
0,5				COMPLETED
1	*Total number of activities= 42*	17	25	COMPLETED
1,5				PENDING
2	*Total number of activities= 27*	17	10	COMPLETED
2,5				PENDING
3	*Total number of activities= 19*	17	2	PENDING
3,5				PENDING
4	*Total number of activities= 10*	10	0	PENDING
4,5				PENDING
5	*Total number of activities= 9*	9	0	PENDING

* Scores in increments of 0.5 show progress along a particular stage.

COUNTRY PROGRESS

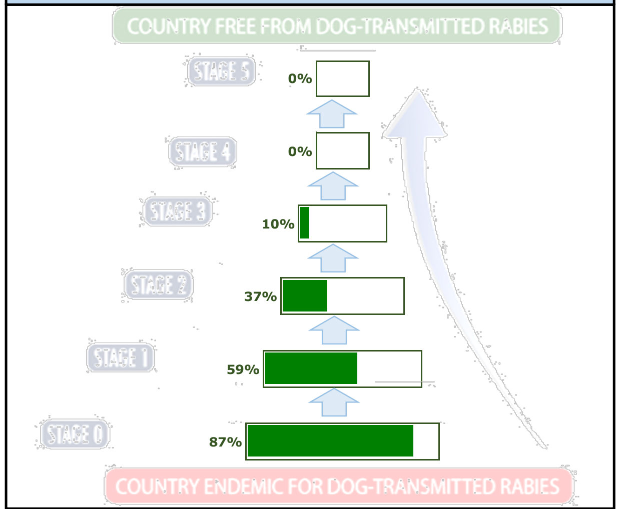

Supplement: Supplementary file 1 [file Data_Sheet_1.pdf]
